# Phylogenomic proof of Recurrent Demipolyploidization and Evolutionary Stalling of the “Triploid Bridge” in *Arundo* (Poaceae)

**DOI:** 10.3390/ijms21155247

**Published:** 2020-07-24

**Authors:** Wuhe Jike, Mingai Li, Nicola Zadra, Enrico Barbaro, Gaurav Sablok, Giorgio Bertorelle, Omar Rota-Stabelli, Claudio Varotto

**Affiliations:** 1 Department of Biodiversity and Molecular Ecology, Research and Innovation Centre, Fondazione Edmund Mach, 38010 San Michele all’Adige (TN), Italy; wuhe@nwafu.edu.cn (W.J.); mingai.li@fmach.it (M.L.); enrico.barbaro@fmach.it (E.B.); 2 Dipartimento di Scienze della Vita e Biotecnologie, Università degli Studi di Ferrara, 44121 Ferrara, Italy; ggb@unife.it; 3 Department of Sustainable Ecosystems & Bioresources, Research and Innovation Centre, Fondazione Edmund Mach, 38010 San Michele all’Adige (TN), Italy; nicola.zadra@guests.fmach.it (N.Z.); omar.rota@fmach.it (O.R.-S.); 4 Department of Biosciences, University of Helsinki, 00014 Helsinki, Finland; gaurav.sablok@helsinki.fi

**Keywords:** *Arundo* genus, chromosome number evolution, adaptive evolution, phylogenomics, molecular dating, polyplodization

## Abstract

Polyploidization is a frequent phenomenon in plants, which entails the increase from one generation to the next by multiples of the haploid number of chromosomes. While tetraploidization is arguably the most common and stable outcome of polyploidization, over evolutionary time triploids often constitute only a transient phase, or a “triploid bridge”, between diploid and tetraploid levels. In this study, we reconstructed in a robust phylogenomic and statistical framework the evolutionary history of polyploidization in *Arundo*, a small genus from the Poaceae family with promising biomass, bioenergy and phytoremediation species. Through the obtainment of 10 novel leaf transcriptomes for *Arundo* and outgroup species, our results prove that recurrent demiduplication has likely been a major driver of evolution in this species-poor genus. Molecular dating further demonstrates that the species originating by demiduplication stalled in the “triploid bridge” for evolutionary times in the order of millions of years without undergoing tetratploidization. Nevertheless, we found signatures of molecular evolution highlighting some of the processes that accompanied the genus radiation. Our results clarify the complex nature of *Arundo* evolution and are valuable for future gene functional validation as well as reverse and comparative genomics efforts in the *Arundo* genus and other Arundinoideae.

## 1. Introduction

*Arundo* is probably the most studied genus in the Arundineae tribe because of its large perennial species with ornamental and economic value. A better understanding of the evolution of the genus *Arundo* is thus relevant to understand how the very high productivity of some of the *Arundo* species originated. Recent taxonomic revisions indicated that up to five species mostly occurring in lowlands and sites affected by human disturbance of the landscape may be included in the *Arundo* genus, namely *Arundo formosana*, *A. plinii*, *A. donaciformis*, *A. micrantha* and *A. donax* [1]. *A. formosana* is an endemic species of Taiwan and the Ryukyu Islands encompassing two subspecies and a total of three varieties [2]. This grass reproduces sexually and it is dispersed through wind via seeds. In its native range, *A. formosana* can be used for protecting hillslopes from soil erosion [3]. *A. plinii*, with a height up to roughly 2 m, is commonly distributed in Italy almost over 1000 km from Bologna (the *locus classicus*, where the species was first described by Pliny the Elder about 2000 years ago) to Sicily and it is present also in Malta, Croatia and Greece. It grows from seed and it is dispersed by wind propagation. Amplified Fragment Length Polymorphism (AFLP) and chloroplast DNA data showed that *A. plinii* is characterized by a large decrease of genetic diversity from the southern part of Italy, to central and northern Italy, where only one ploidy level exists (*12x* uniform cytotype). However, haplotype diversity has been detected in both the south and north of the Balkans [1,4]. In addition to genetic diversity, also variations in chromosome number characterize *A. plinii*. In southern Italy populations with two ploidy levels exist: a cytotype with 72 chromosomes, corresponding to a putative dodecaploid chromosome number of *2n* = *12x*, and a second cytotype with 108 chromosomes, corresponding to a putative octadecaploid chromosome number of *2n* = *18x*. Based on the lack of a clear distinction of both morphometric and molecular data, it has been recently proposed that *A. collina* (until recently considered a separated species [5,6] should be included in *A. plinii* [4]. *A. donaciformis* is a polyploid species (*2n* = 108) with asexual reproduction, it occurs mainly in southern France and northwest Italy and plays an important role in preventing soil erosion through its powerful rhizomes. *A. donaciformis* is closely related to *A. plinii*, with whom it shares all chloroplast haplotypes available to date. Thus, it has been proposed to have originated from *A. plinii* by pseudotriploidization [4]. *A. micrantha* is distributed in the Mediterranean region and North Africa along rivers and streams. This species of grasses is endangered by the competition of another invasive species from the same genus, *A. donax* [1,7]. 

*A. donax* is one of the most promising bioenergy crops among rhizomatous grasses together with *Phalaris arundinacea*, *Miscanthus* and switchgrass [8]. *A. donax*, also called “giant cane” or “giant reed” is a perennial grass with C_3_-type photosynthesis. It is an octadecaploid species (*2n* = *18x* = 108–110) sharing with *A. micrantha* complete sterility due to early failure of both male and female gamete development [9,10]. Genetic studies indicated that *A. donax* geographically originated in Eastern Asia, from where it spread all around the world, possibly by human intervention for different purposes [11]. Several varieties of *A. donax* have been described, sometimes with putatively lower chromosome numbers, like e.g., *A. donax* var. *macrophylla* (*2n* = 40 [8]), which has large glaucous leaves and shorter culms. *A. donax* requires little management input and lacks natural competition, which, in addition to great adaptability, makes it at the same time an invasive grass and a valuable bioenergy crop [12]. Introduced in the USA in the early 1800s for erosion control [13], it quickly spread to southern US becoming invasive by outcompeting native plant species, reducing wildlife habitats and negatively impacting river hydrology [14]. These features make it a very productive biomass species, as in the right environment *A. donax* fields become productive from the second year and produce high dry biomass yields up to roughly 40 tons/hectare/y [15], corresponding to an energetic value of about 150 MWh/hectare/y. 

Polyploidy and chromosome number changes are considered as important drivers of speciation both in animals and especially in plants, and many crops are relatively recent polyploids [16]. The most common mechanisms of polyploidization are whole-genome duplication (leading to doubling of the original chromosome number) and triploidization (more generally called demiploidization or demipolyploidization, or the increase of the chromosome number by 50% of the original number). Triploidization is often considered a transient phase (“triploid bridge”) towards chromosome doubling [17]. Triploids are widely acknowledged to originate from gametic “nonreduction” (also called “meiotic nuclear restitution”) of micro- and megaspores, thus leading to the production of a proportion of unreduced gametes having the full somatic chromosome number [18]. Chromosome number evolution and polyploidization have attracted great attention in plants [19,20]. Polyploids and their ancestors usually differ in morphological, physiological and evolutionary life-history traits [21], and these differences have been repeatedly linked to the enhanced ability of polyploid species to adapt to new environments [22]. In the Poaceae family, different clades experienced polyploidization via hybridization (e.g., Bamboos [23]). Assuming a base chromosome number x = 6 [10], *Arundo* species have two major cytotypes: *2n* = *6x* = 72 (*A. formosana*, *A. plinii*, *A. micrantha* and Asian *A. donax* accessions [1,4]) and *2n* = *9x* = 108 (*A. donax*, *A. donaciformis* and some populations of *A. plinii* [4,11]), indicative of repeated polyploidization (triplodization). The possible origin of *A. donax* invasive clone and *A. donaciformis* by polyploid speciation from lower ploidy levels *A. donax* Asian accessions and *A. plinii*, respectively, has been proposed [4,11]. Additional proposals about the origin of *A. donax* via autopolyploidization from *A. plinii* or allopolyploidization involving *A. plinii* and *P. australis* as parental species [24] are considered less likely [10], but none of these hypotheses up to now has been formally tested in a statistical framework. A deeper understanding of the dynamics of chromosome evolution could be relevant to assess potential strategies for *Arundo* improvement based on the induction of ploidy level variations.

The extraordinary vigor of *A. donax* does not seem to be explained alone by its ploidy level, as several traits like plant height and rhizome size only partially correlate with chromosome numbers within the genus [1]. Comparative characterization of the patterns of molecular evolution and especially the identification of candidate genes under positive selection can potentially provide important clues on the genetic bases of such differences and their adaptive relevance. Previous studies of orthologous plant datasets derived from transcriptomes have inferred positive selection (e.g., [25]), but to date, no such approach was carried out in the *Arundo* genus.

Reconstructions of both chromosome numbers and patterns of natural selection rely on a robust species phylogeny of the taxa object of study. The most comprehensive dated phylogeny of the *Arundo* genus obtained so far estimated its crown age at around 10 million years ago (MYA), placing *A. donax* as the most basal taxon within the genus [4]. Being based on only five plastid spacer regions (which are maternally inherited), this phylogeny represents a chloroplast gene phylogeny and as such it may be unsuitable to reliably reconstruct the evolutionary history of the genus owing to the frequent ploidy changes and the possibility of hybridization [24].

In this study, we generated, assembled and characterized the leaf transcriptomes of seven *Arundo* taxa/accessions and three related outgroups from the Arundinoideae subfamily of Poaceae. We then reconstructed a nuclear-based species phylogeny of the *Arundo* genus using a phylogenomic approach, carried out a molecular dating of the age of the different clades, and finally investigated chromosome number changes within the *Arundo* genus in a statistical framework. Understanding the evolution of this important genus of grasses lays the foundations for future comparative genomics studies. In addition, it may help to support ongoing efforts to establish reverse genetics and functional genomics approaches in *A. donax*, thus contributing to providing promising candidate genes for the improvement of this biomass species.

## 2. Results

### 2.1. Ten New Transcriptomes for Arundo and Arundinoideae Taxa

De novo assembly of reads from all known *Arundo* species and three outgroups yielded in total 1,016,877 unigenes with average length ranging from 740 to 1064 bp (Table S1). The N50 of the assembly ranged from 1432 and 1850 bp, with a max transcript length between 10,952 bp and 15,976 bp (Table S1).

Similarity searches of assembled unigenes carried out against the *A. thaliana*, *S. italica* and the NCBI nonredundant (Nr) protein databases with FunctionAnnotator resulted in 12.4–24.0%, 17.7–35.2% and 58.6–74.7% unigenes with significant hits, respectively. The assigned unigene functions (Table S2) covered a broad range of gene ontology (GO) categories corresponding to 671,330 transcripts (66.02%). All 10 transcriptomes had similar distributions of GO terms, thus we aggregated the data to provide more detailed statistics on the resulting Arundinoideae leaf metatranscriptome (Table S3). Under the molecular function category, 45.1% of the unigenes were associated with binding and 40.7% of the unigenes had catalytic activities. Among the binding and catalytic activities category, ATP binding represented the most abundant classification, followed by ion binding, DNA binding and protein serine/threonine kinase activity. The biological process category showed that 20.3% of the genes were associated with metabolic process, 19.7% with cellular process and 8.6% with response to stimulus. The cellular component category showed that genes associated with the cell and cell part were 27.1% and 27.1%, respectively, while 22.9% were associated with organelle and 9.0% with organelle part. The functional annotation of the unigenes was also performed by sequence similarity searches against the *A. thaliana* and *S. italica* proteins using the BLASTx algorithm. A total of 172,745 (16.99%) unigenes functional hits corresponded to *A. thaliana*, and a total of 256,783 (25.25%) unigenes functional hits corresponded to *S. italica*.

### 2.2. A Nuclear Phylogenomic Reconstruction of Arundo Species Reveals A. Formosana as Basal and A. Micrantha as Likely Hybrid

**Figure 1:**
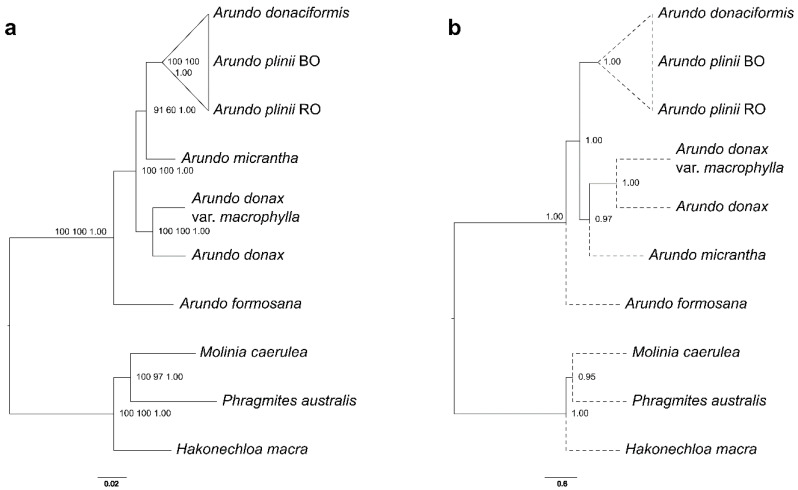
Species tree reconstructions of the *Arundo* genus from the dataset of 144 nuclear genes. (**a**) maximum likelihood (ML) topology of the species tree performed by IQ-TREE using the edge-linked optimal partition scheme of the gene supermatrix. Numbers are ML ultrafast bootstrap (percentage), maximum parsimony (MP) non parametric bootstrap (percentage) and posterior probability (fraction) support values. (**b**) Coalescent reconstruction of the species tree performed by ASTRAL III using the supertree approach on the ML trees of each gene. Dotted lines represent topological connections as ASTRAL does not estimate branch lengths for terminal branches. Numbers are local posterior probabilities for the main quartet topology.

A total of 144 nuclear unigenes that were putative one-to-one orthologs among the 10 species/accessions, were used for maximum likelihood (ML), maximum parsimony (MP) and Bayesian inference (BI) phylogenomic reconstruction of the species tree for the *Arundo* genus. The supermatrix and the annotation of the orthologous groups constituting it are provided in Supplementary File 1 and Table S4, respectively. This dataset contained 1,795,170 nucleotides in 179,517 ungapped aligned columns (corresponding to 59,839 codons, of which 8901 formed distinct patterns, 10,465 were parsimony-informative, 6683 singleton sites and 42,691 constant sites). All but two of the branches in ML, MP and BI phylogenomic reconstructions received high statistical support. The basal species of the *Arundo* genus and sister to all others was *A. formosana* with full bootstrap support (Figure 1). 

As expected, the two *A. donax* varieties, *A. donax* and *A. donax* var. *macrophylla*, clustered together. *A. plinii* accessions formed a highly supported cluster but they could not be resolved from *A. donaciformis* (Cartoonized clade in Figure 1a). The other group which did not receive full support is the one grouping *A. micrantha* with the *A. plinii*/*A. donaciformis* clade (UFBOOT 91%, MP 60%). The BI inference tree obtained by BEAST2 is topologically identical to the ML and MP trees, and all branches are fully supported (all posterior probabilities = 1).

The species tree inferred by a coalescent supertree approach had overall high local posterior probabilities (Figure 1b). The topology was consistent with that of the ML supermatrix tree in having *A. fomosana* as sister to all other *Arundo* species, a strongly supported cluster encompassing the two *A. donax* varieties and *A. plinii* not resolved from *A. donaciformis*. However, the coalescent species tree indicated *A. micrantha* as sister to the clade of *A. donax* varieties. 

**Figure 2:**
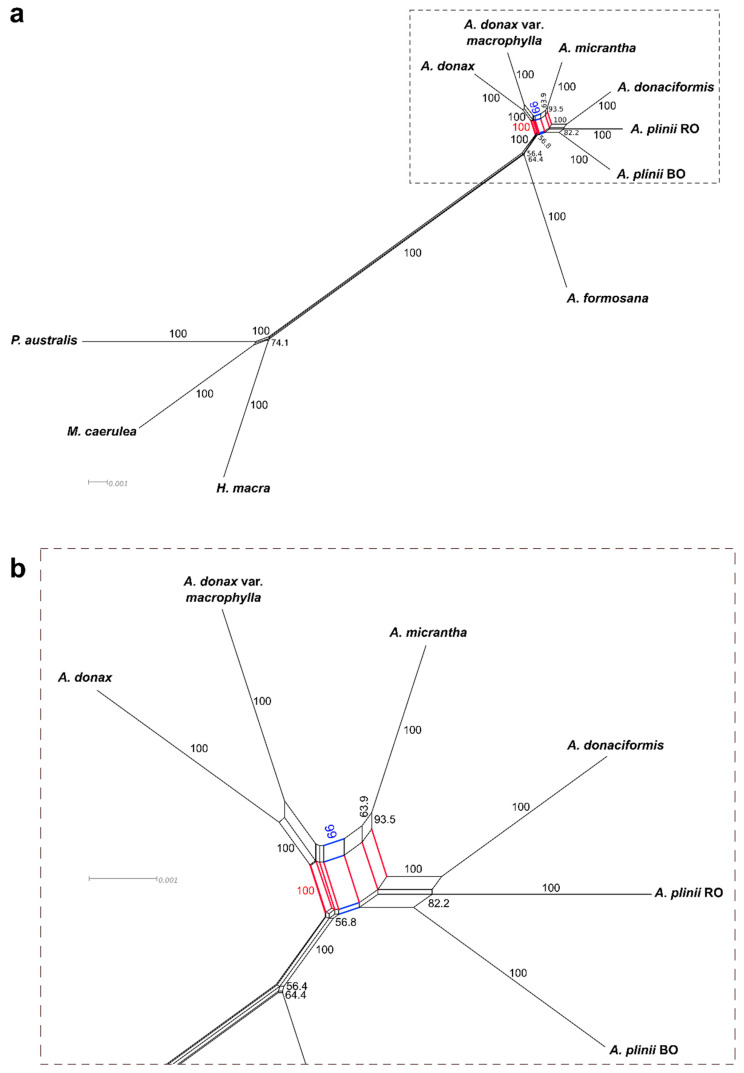
Phylogenetic network of the *Arundo* genus from the nuclear dataset. Values are bootstrap support for each split. Only values above 50% are shown. Branches and bootstrap values in red indicate split support for the grouping of A. micrantha with A. donax varieties, while branches and bootstrap values in blue indicate split support for the grouping of A. micrantha with the A. plinii s.l. clade. (**a**) Complete network. (**b**) Detail of the part of the complete network contained in the dotted rectangle.

We carried out additional analyses to pinpoint sources of phylogenomic incongruence in our nuclear dataset between ML/MP/BI supermatrix trees and the coalescent supertree in the *Arundo* lineage. First, we estimated concordance among genes and sites of the ML supermatrix tree. Gene concordance factor (gCF), a measure of phylogenetic concordance among genes, had low values for the majority of branches, indicating conflicts among the single-gene trees. While the overall pattern of gCF and site concordance factor (sCF; a measure of phylogenetic concordance among sites irrespective of the genes they belong to) support was consistent across branches, highly significant differences between the two indices could be detected for the branch separating *A. micrantha*/*A. plinii* s.l. from *A. donax*/*A. formosana* at both genes and sites level (see the probability of equal frequencies rejection for genes, gEF_p, and for sites, sEF_p, for branch 13; Table S5), indicating that the low concordance values for this branch were not due to incomplete lineage sorting [26]. gCF and sCF indicated that two of the branches (11 and 13, defining the topological placement of *A. donaciformis* and *A. micrantha*; Gene Internode Certainty, gIC, and Site Internode Certainty, sIC, in Table S5) had discordance factors for the first alternative topology higher than the concordance factors for the given topology. These two branches were the shortest in the whole phylogeny, indicating that the lack of sufficient and/or conflicting phylogenetic information caused the observed incongruence. Branch 13 had significantly different values for both gene discordances and site discordances indices, and the lowest gene and site internode certainties, suggesting that the given and the first alternative topology were both highly supported (Table S5). Accordingly, network analysis uncovered extensive topological incongruence especially for *A. micrantha* (branch 13), providing equal bootstrap support to the grouping of the species with either *A. donax* varieties or the *A. plinii*/*A. donaciformis* clade (Figure 2). 

A very similar number of characters supported each alternative split, but the majority of genes supported the grouping of *A. micrantha* with *A. donax* (Table S6). Repetition of the analysis on the partition constituted only by first and second codon positions to reduce the number of ambiguous characters (i.e., those in support of both hypotheses because of homoplasy) also indicated a slightly closer relationship between *A. micrantha* and the *A. donax* varieties (Table S6). A large portion of characters, however, still supported clustering of *A. micrantha* with the *A. plinii*/*A. donaciformis* clade.

Additionally, analysis of alternative quartet topologies of the coalescent supertree (normalized quartet score: 0.668) indicated that quartet topologies around *A. donaciformis* and *A. micrantha* had the lowest difference among the main topology and the two alternative topologies (Table S7), confirming these two taxa as a major source of incongruence among single-gene trees and further suggesting the possible hybrid origin of *A. micrantha*.

### 2.3. Extended Plastidial Dataset Confirms Basal A. Formosana and Supports Parphyletic A. Donax

**Figure 3:**
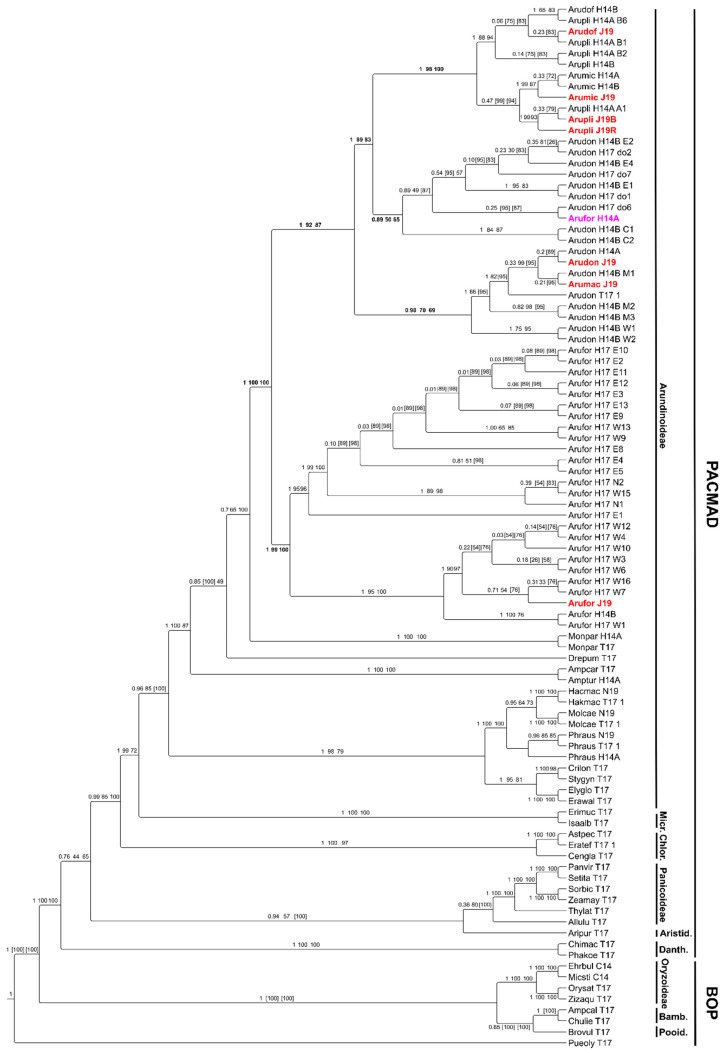
Combined ML and Bayesian inference (BI) cladogram of the Arundinoideae and selected species from PACMAD and BOP clades based on 5 chloroplast intergenic regions. Taxa in red are those used in this study for leaf RNA-Seq. The taxon in purple corresponds to the misidentified *A. formosana* accession used in Hardion et al. 2014. Branch supports are poster support from BI analysis (first number) and ultrafast bootstrap support from ML analysis (second number). The reference topology is obtained by BI. Numbers in square brackets indicate the support for the alternative ML topology. Cladogram drawn with Tree Graph 2.

The chloroplast dataset encompassed a total of 95 species, and five chloroplast loci/species with 4308 aligned columns, 2346 distinct patterns, 1074 parsimony-informative, 862 singleton sites and 2372 constant sites (the subset of 10 species used in RNA-Seq had 4308 columns, 99 distinct patterns, 120 parsimony-informative, 69 singleton sites, 4119 constant sites). The supermatrix and the annotation of the sequences constituting it are provided in Supplementary File 2 and Table S8, respectively. Phylogenetic reconstruction of this maternal dataset by ML (tree provided in Supplementary File 3), MP or BI inference confirmed the basal position of *A. formosana*. It also grouped *A. micrantha* with a polyphyletic *A. plinii*/*A. donaciformis* clade with high statistical support (BI PP = 1; ML UFBOOT = 98%; MP npBS = 100%), suggesting this as maternal lineage of *A. micrantha* putative hybrid. According to previous work [11], *A. donax* was not monophyletic in our plastid phylogenies (Figure 3): the clade comprising *A. plinii* was nested within paraphyletic *A. donax.* The clade of the *A. donax* invasive clone was basal to that of Asian *A. donax* accessions. As expected, monophyly of *A. formosana* was instead well supported [2]. All our samples used for RNA-Seq are correctly clustered with the corresponding species. The *A. formosana* accession used for the chronogram in [4] clustered with the Asian clade of *A. donax*, but was separated from all other *A. formosana* accessions, indicating taxonomic misidentification (Hardion pers. comm.). 

### 2.4. Molecular Dating of Arundo Origin

**Figure 4:**
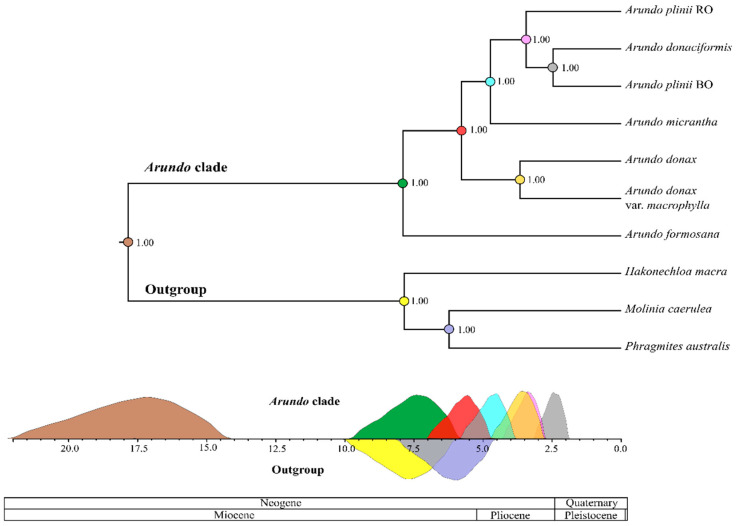
Chronogram of the *Arundo* genus based on the set of 144 nuclear genes. Numbers close to nodes indicate posterior support. The scale under the tree is in million years and the 95% CIs are drawn in the color of the node they correspond to in the chronogram. The 95% CI for the *Arundo* ingroup is above the scale and under the scale for outgroups.

We estimated the divergence of *Arundo* species using both the nuclear and the plastid datasets. Model selection indicated that the nuclear dataset is best fitted by a combination of Yule demographic prior and a lognormally distributed relaxed clock (Table S9). The summary of the clock rates estimated from the nuclear dataset with different models and data partitions are provided in Table S10. The Bayesian consensus tree for the most fitting model in the nuclear dataset is reported in Figure 4, while in Table S11 we report the mean age for each of the nodes, together with the corresponding 95% High Posterior Densities (HPD, a type of confidence interval). According to Figure 4, all the posterior estimates for the age of nodes are characterized by a normal distribution which is indicative of good convergence of the analysis. The origin of the *Arundo* genus is set at 17.8 MY (mean estimate) with HPD ranging from circa 14 to 22 MY. The diversification of the extant *Arundo* started circa 7.9 MY (6.2–9.8 HPD), which corresponds to the split of *A. formosana*. The nuclear estimate for the diversification of extant *Arundo* is compatible with divergence estimates based on plastidial data (mean 9.6 MY, HPD 7.6–11.5 MY, Figure 4) particularly because there is a large overlap of posterior densities (Figure S1; Table S12). 

The lineage leading to *A. donax* diversified circa 5.7 MYA and the split of the two varieties of *A. donax* sampled in this study is placed at 3.7 MYA, thus providing a conservative estimate of the time required for the generation of morphological differentiation within this clonal species. Divergence of *A. micrantha* is estimated at 4.7 MYA. Finally, the divergence of the *A. plinii* s.l. clade took place around 3.4 MYA, followed by the split of *A. donaciformis* from *A. plinii* around 2.5 MYA. 

### 2.5. Demiduplication Is the Most Prominent Mechanism of Chromosome Number Evolution in Arundo

As a species tree for the reconstruction of chromosome evolution, we used the maximum clade credibility (MCC) tree obtained with BEAST2 pruned to assess only one representative accession per species. ChromEvol analyses determined that among the 10 alternative evolutionary models tested, the model supporting four types of transitions between chromosome numbers (constant rates of ascending and descending dysploidy, duplication and independently estimated demipolyploidization; CONST_RATE_DEMI_EST, Table 1; Table S13) was the best-fitting model of the process of chromosome evolution in *Arundo*. 

**Figure 5:**
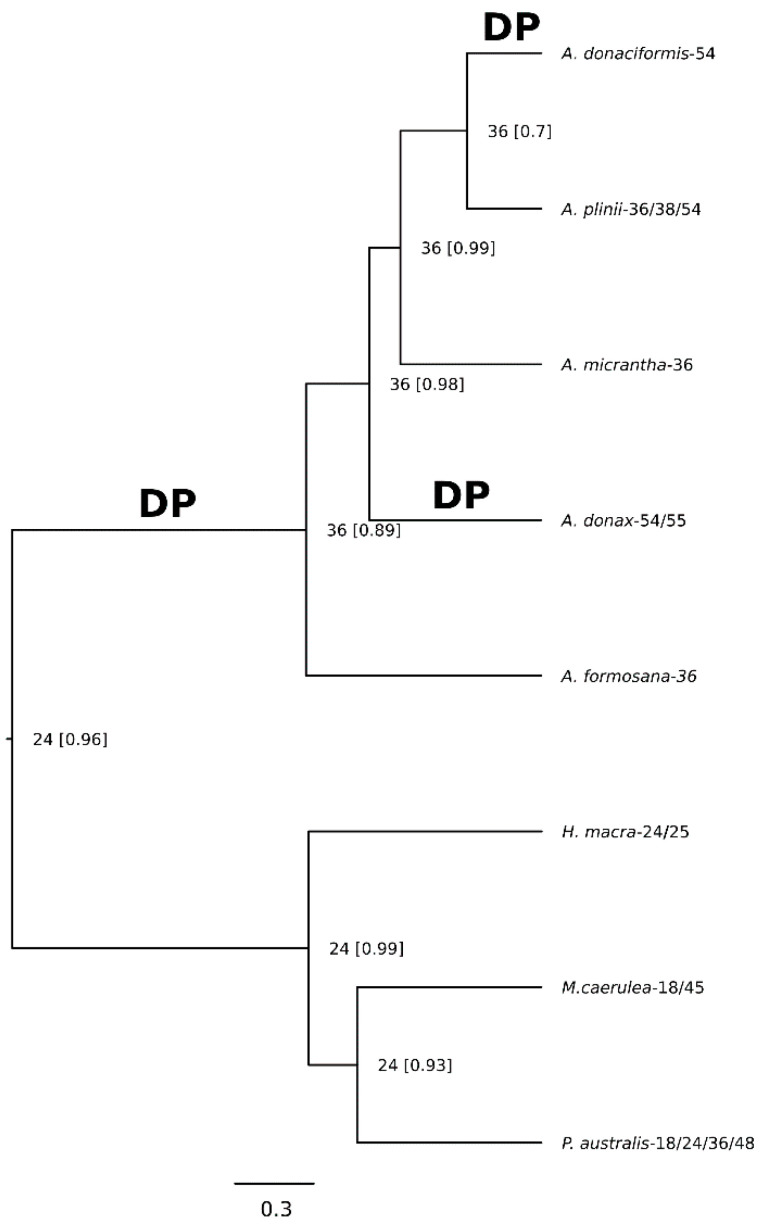
Chromosome number evolution and inferred ancestral chromosome state in the genus *Arundo*, including three outgroups, inferred under Bayesian optimization. For each node, the most probable ancestral haploid chromosome number is reported. Numbers in square brackets are posterior probabilities. Numbers at the tips are the most frequent known haploid chromosome numbers of each species. DP: inferred demipolyploidization event.

In general, models where the rates of transition are dependent on the starting chromosome number of each transition have largely worse fits than those with constant transition rates over the whole phylogeny. Worthy of noting, despite having an additional parameter, the best model has a better AIC score compared to its sister model with equal rates of duplication and demipolyploidization, indicating a much higher rate of demiduplication than whole-genome duplication (Table S13). The evolutionary history for chromosome number changes inferred by ChromEvol for the *Arundo* genus is shown in Figure 5. The asterisk (*) indicate the best model.

The haploid chromosome number inferred for the ancestor of all *Arundo* species was *n* = 36 (posterior probability (pp) = 0.89). The same haploid chromosome number was maintained along the whole backbone of the phylogeny indicating that all extant *Arundo* species evolved from ancestors with *n* = 36 chromosomes. The haploid chromosome number inferred for the branch leading to *A. micrantha*, *A. plinii* and *A. donaciformis* was *n* = 36 (pp = 0.98), while in the case of *A. plinii* and *A. donaciformis* branch the haploid chromosome number was *n* = 36 (pp = 0.99) and increased to *n* = 54 in *A. donaciformis* through a demiduplication event. The inferred ploidy level of *Arundo* taxa showed that all of them are polyploid species based on the inferred root haploid chromosome number *n* = 24 (pp = 0.96; Table S14). Under the most recent common ancestor haploid number *n* = 36, demiduplication clearly results to have been the main driving force in chromosome evolution for the *Arundo* genus.

### 2.6. Patterns of Molecular Evolution Identify Candidate Genes under Positive Selection in Arundo and Arundinoideae

A total of 22 genes with evidence of positive selection (*p* < 0.05) were identified. Among them, seven genes were still significant after multiple test correction (at 5% FDR; Table S15). The putative functions of these genes, annotated by BLAST similarity searches against *Arabidopsis thaliana*, are reported in Table 2. 

We then tested whether specific branches of the phylogeny could be affected by positive selection. Among the 144 orthologous groups, two did not produce any output. Of the remaining 142, a total of 14 genes and 17 branches with evidence of positive selection (corrected *p*-value < 0.05) were identified. The only two orthologous groups with more than one positive branch were OG0018357 (2 branches) and OG0018377 (3 branches), both of which were under pervasive diversifying selection also according to BUSTED analyses. Only terminal branches resulted positive, with the branches of *H. macra*, *P. australis*, *A. micrantha* and *A. donax* var. *macrophylla* hit only once, and the branches of *A. donaciformis* and *M. caerulea* hit each twice (Table S16). When tested specifically for selection relaxation/intensification, the majority of the genes (12 out of 14) showed K values higher than 1, indicative of selection intensification. Of the 14 genes tested, only seven genes remained significant after multiple test correction (at 5% FDR; Table 3) and all of them showed evidence for selection intensification along the branches identified by ABSREL (K > 1; Table S16). 

The gene with the highest evidence of selection intensification was OG0018069 (branch of *A. donaciformis*, K = 39.10), followed by OG0018205 (branch of *M. caerulea*, K = 9.25; (Table S16)).

Out of the 144 genes analyzed, only one (OG0017482) was predicted to be neither under pervasive purifying nor directional selection at the codon level. All other 143 genes were found to contain 8% of total codons subject to pervasive purifying selection with posterior probability larger than 0.9, while only 26 genes contained 0.16% of total codons subject to pervasive purifying selection. A total of 56 genes out of the 144 analyzed were found to contain a total of 99 sites displaying signatures of episodic diversifying selection with *p* > 0.05 (0.17% of total codons in the dataset). Out of these 99 sites, 14 high-confidence codons from 12 genes were identified by two approaches (Fast Unconstrained Bayesian Approximation (FUBAR) and Mixed Effects Model of Evolution (MEME)) as under purifying selection (Table S17). Two of the codons remained significant after conservative Sime’s correction for multiple testing: codon 594 of OG0018357 (*q* = 0.0022) and codon 890 of OG0018374 (*q* = 0.0430).

## 3. Discussion

In this study, the leaf transcriptomes for all recognized taxa of the *Arundo* genus and three Aundinoideae outgroups were generated and annotated, allowing the first nuclear phylogenomic reconstruction of the *Arundo*, which up to now was based only on plastidial datasets [2,4].

### 3.1. A New Scenario of Arundo Phylogeny

Our nuclear phylogenomic reconstructions with ML, MP and BI approaches differ from those earlier chloroplast phylogenies in having *A. formosana* instead of *A. donax* as the basal species of the *Arundo* genus. The comprehensive chloroplast phylogeny reconstructions with representatives of the BOP and PACMAD clades we carried out with both ML and BI methods include all *Arundo* sequences formerly published. Both phylogenies clearly show that the *A. formosana* accession used in our study is monophyletic to all other *A. formosana* accessions with the exception of the one used in [4], which is instead nested in the Asian clade of *A. donax* accessions. This evidence demonstrates that the correct placement of *A. formosana* is at the base of the genus and rules out that the observed incongruence among studies could stem from differences in genome type and parent of origin [27,28,29]. The basal position of *A. formosana* strongly supports the origin and diversification of *Arundo* in Eastern Asia during the Miocene. This timing predates the tectonic event that led to isolation of Taiwan island, suggesting that interspecific competition with other *Arundo* lineages may have at least in part contributed to vicariance of *A. formosana* to Taiwan and the Ryukyu islands [2]. Additionally, it indicates that future efforts of comparative genome sequencing in the *Arundo* genus should include *A. formosana* as reference species. According to our results, the evolutionary lineage of the *A. donax* invasive clone originated between the late Miocene and the early Pliocene, and possibly predates the radiation of the *A. donax* eastern clade, confirming *A. donax* invasive clone as one of the most ancient and invasive super-genotypes known [11,30]. The chloroplast phylogenetic analyses, also, consistently suggest that *A. donax* var. *macrophylla* is most likely a variety with larger leaves of the invasive clone of *A. donax* present in the Mediterranean area, rather than an Asian accession as suggested by previous results [13]. 

### 3.2. Hybridization Events Clarified 

Previous studies proposed different scenarios for the evolutionary origin of *A. donax* invasive clone, including *A. plinii* autopolyploidization and allopolyploidization between *A. plinii* and *P. australis* [24]. Our results conclusively rule out both hypotheses as *A. donax* is basal to *A. plinii* and its nuclear genome does not show any signs of introgression from *P. australis*. Interestingly, however, our nuclear data suggest that *A. micrantha* is possibly the result of hybridization between the *A. donax* and *A. plinii* lineages. The chloroplast phylogeny places *A. micrantha* in the *A. plinii* s.l. clade, in line with previous results [4]. The different topologies of *A. micrantha* in nuclear and chloroplast phylogenies are consistent with a cross between a mother plant from the *A. plinii* lineage and a father plant from the *A. donax* lineage. *A. micrantha* and *A. donax* share much lower percentages of viable pollen compared to *A. plinii* and *A. donaciformis* and no germination in the conditions tested until now [10]. Early failure of micro-gametogenesis in both *A. micrantha* and *A. donax* is associated to cytomixis, which is not apparent in other *Arundo* taxa [10], indicating that *A. donax* invasive clone is most likely not the parent of origin of *A. micrantha*. More likely, *A. micrantha* appears to be the result of an extremely rare hybridization event that took place during the evolution of *A. donax* and *A. plinii* lineages, maybe involving a fertile individual from the lower ploidy lineage of Asian *A. donax*. 

### 3.3. Recurrent Demipolyploidization in the Arundo Genus Does Not Lead to Tetraplodization

Besides this isolated hybridization event, the analysis of chromosome evolution we carried out demonstrates in a statistically robust framework that demiduplication, together with the ability to propagate vegetatively, played a major role in the evolution of the *Arundo* genus. The base number we used in chromosome evolution analysis (*x* = 6, the most probable among the alternatives; Ref. [10] indicates *n* = 24 as the most likely haploid chromosome number for the ancestors of the *Arundo* genus, but this result will require further confirmation when a more complete record of chromosome counts for Arundineae and Molinieae becomes available (CCDB [31])). Based on the data currently available, demiduplication is predicted to have taken place three times independently in the *Arundo* lineage: (1) once before the diversification of the most basal *Arundo* species sampled in this study, *A. formosana*, in the lapse of time between 17.8 MYA and 7.9 MYA. This brought the chromosome number of all branches ancestral to extant taxa to *2n* = *12x* = 72; (2) a second time during the evolution of *A. donax* invasive clone (*2n* = *18x* = 108), between 5.7 MYA and 3.6 MYA; and (3) a third time in the lineage of *A. donaciformis* (*2n* = *18x* = 108), about 2.4 MYA. The observation that this is still an ongoing process in *A. plinii* populations [4] further corroborates the fundamental role played by demiduplication throughout the evolution of the *Arundo* genus. All *Arundo* species are perennials and can propagate by vegetative reproduction, both characters that facilitate the establishment of stable populations with triploidized *18x* cytotypes [32,33]. These factors can possibly explain the recurrent phenomenon of demiploidization in different *Arundo* lineages and theoretically provide ample opportunity for the appearance of cytotypes functionally corresponding to tetraploids from *18x* cytotypes according to the widely accepted triploid bridge model of polyploidy evolution [18]. Based on our estimations, the origin of these taxa dates back to several million years ago, but, despite this relatively long evolutionary time, no cytotype was found that results from the doubling of the basal chromosome number (*2n* = *12x* = 72) of the genus. This is easily explained in the case of the Mediterranean *A. donax*, given its sterility [9,10]. By contrast, the *A. plinii* s.l. lineage would seem particularly suited to the evolution of new *24x* populations, given the high number of *18x* cytotypes identified so far [4]. Field observations suggest that self-incompatibility may partly explain the lack of seed development in *18x* cytotypes in natural populations, but this reproductive barrier seems to be relatively labile, as seed set was observed in common-garden experiments with different accessions [10]. In general, despite being to some extent species-specific, the distributions of progeny cytotypes in crosses between triploid and diploid individuals are biased towards diploids, often with complete absence of tetraploids and triploids [18]. Thus, the lack of tetraploid-like *24x* cytotypes in *Arundo* may be at least partly due to exclusive production of *12x* progeny from crosses between *18x* and *12x* cytotypes in mixed populations. Another nonmutually exclusive possibility is that the high number of chromosomes in *Arundo* species may represent an upper limit to further duplication: Although Poaceae species with higher chromosome numbers are known (e.g., *n* = 133 in *Poa litorosa* Cheeseman, [34]; *n* = 126 in *Helictotrichon alpinum* (Roem. and Schult.) Henrard, [35]; *n* = 108 in *Echinochloa stagnina* (Retz.) P. Beauv. [36]), the *18x* cytotype (2*n* = 108) of *Arundo* species ranks among the 2% highest chromosomal numbers in Poaceae and only 10 among more than 4200 species surveyed are reported to have ≥ 144 chromosomes, which would correspond to a *24x* cytotype [31]. We, thus, propose that, in *Arundo*, chromosome number duplication may negatively affect plant fitness by contrast to demiduplication, which seems to represent the natural upper limit of chromosome numbers in this lineage.

### 3.4. Implications for Genetic Improvement in Arundo

Based on the evolutionarily-informed observations outlined above, future strategies for *Arundo* improvement based on doubling of ploidy levels may be less effective than in *Mischanthus* x *giganteus*, an allopolyploid biomass species with *2n* = *3x* = 57, where hexaploid plants were obtained albeit without significant increases in biomass yield-related traits [37]. On the other hand, the phenotypic variation of natural or experimentally-generated *18x* cytotypes of *A. plinii* may not be sufficient to match the extraordinary productivity of *A. donax* [4], which, in line with a possible eastern *A. donax* ancestry of *A. micrantha*, may require the contribution of the Asian gene pool. The recent development of a mutant population [38] could represent a valid alternative for the straightforward introduction of phenotypic variation in *A. donax*. The transcriptomics resources developed in our study, and in particular the candidate genes for positive selection, could be a convenient starting point for reverse genetic approaches in this species, as they could regulate its ability to grow in marginal soils. While for gene-wide positively selected candidates (BUSTED analyses) the species originating the signal are not known, the three genes with signatures of selection intensification along *A. donaciformis* and *A. donax* var. *macrophylla* branches have a potentially adaptive value. One of those, OG0018204 is predicted to encode the nuclear core subunit of NADH dehydrogenase (ubiquinone) flavoprotein 1, a protein of the mitochondrial respiratory chain reducing ubiquinone by NADH [39], suggesting that adaptive variations in oxidative phosphorylation efficiency could have taken place during the evolution of *A. donaciformis* following demipolyploidization. The strongest evidence of selection intensification in *A. donaciformis* was, however, found in OG0018069, a homolog of BIP2, an Arabidopsis heat shock 70 kDa protein localized in the ER lumen [40]. In Arabidopsis BIP2 and its paralog BIP1 control fusion of polar nuclei in female gametophytes, as well as endosperm development and embryo viability [40], suggesting that in *A. donaciformis* OG0018069 may have undergone adaptive selection contributing to overcome the triploid block to seed development reported in many species and usually associated to abnormal endosperm development [18,41]. Thus the patterns of diversifying selection observed in *A. donaciformis* OG0018069 could at least in part underlie the ability of this triploid species to set seeds at rates comparable to those of *A. plinii* [10]. Another interesting candidate gene which could have played a relevant role in the evolution of *A. donax* is OG0018357. The Arabidopsis homolog of this gene, *CER9*, is a RING-type E3 ubiquitin transferase involved in cuticular wax biosynthesis regulation through post-transcriptional enhancement of 3-hydroxy-3-methylglutaryl-coenzyme A reductase activity, the major rate-limiting step of the mevalonic acid pathway [42,43]. Compared to the most common *A. donax* invasive clone, *A. donax* var. *macrophylla* has glaucous leaves, which could be related to altered cuticular wax amount/composition, but differences in cuticle compositions have not been analyzed so far. OG0018357 is overall the gene with the highest evidence of natural selection in our dataset, being positive to all methods we applied to determine positive selection. As *CER9* is involved also in water use efficiency and drought resistance [42,43], future functional studies should address whether OG0018357 contributed to the adaptation of *Arundo* species/varieties to water stress. 

## 4. Materials and Methods

### 4.1. Transcriptome de Novo Assembly

A total of 10 species/accessions of Arundinoideae were used, seven from the *Arundo* genus and three from related Arundinoideae outgroups (Table S1). Total RNA was extracted from leaves of all species/accessions, converted to cDNA and sequenced with 100 bp paired-end Illumina reads with an Illumina HiSeq2000 sequencer according to [44]. The raw reads are deposited in GenBank under project PRJEB36611 with accession numbers ERR3891548-ERR3891557. Quality assessments were conducted on these raw reads by FastQC v0.11.9 (https://www.bioinformatics.babraham.ac.uk/projects/fastqc//). The Bowtie index was constructed with the bowtie-build utility from Bowtie v. 1.0.0 [45] using the Enterobacteria phage phiX174 fasta file as reference. Adapters were cleaned by mapping of the reads to the phiX genome. Sequence adapters and low-quality regions reads were trimmed by Trimmomatic v. 0.32 [46], the reads with length ≥ 100 bp were retained. Properly paired reads were found with the fastqutils properpairs from NGSUtils v. 0.5.9 [47]. Transcripts were de novo assembled using the Trinity Assembler v2.11.0 [48] with default settings. To lower the redundancy in the dataset, the transcripts were clustered using the CD-HIT-EST v4.8.1 program [49] with an identity threshold of 95% and a word size of 10. The CAP3 [50] tool was used to generate unigenes for each cluster with default options and an identity of 99%.

### 4.2. Gene Functional Annotation

Assembled unigenes were annotated by performing a local BLASTx search against the *Arabidopsis* TAIR10 database (https://www.arabidopsis.org/) and all *Setaria italica* proteins from the UniProt database (http://www.uniprot.org/) with an E-value cutoff of 1 × 10^−10^. Gene ontology annotation was carried out using the web-based program FastAnnotator (http://fa.cgu.edu.tw/index.php) [51].

### 4.3. Orthologous Groups Identification and Supermatrix Construction

The TransDecoder v. 2.0.1 [52] program was used to identify the candidate open reading frames (ORFs) within the transcripts with default parameters, and sequences with a protein length greater than 100 were retained for further analysis. The obtained ORFs were used for local BLASTp search against the protein database of *Setaria italica* with a cutoff e-value of 1 × 10^−5^, and all hits were retained as putative coding regions. Translated sequences were clustered into multiple orthologous groups of all species/accessions using OrthoFinder 2.4.0 [53] with default parameters. 

The phylogenetic supermatrix was reconstructed based on putative 144 one-to-one orthologous groups (OGs) among all 10 species. Single orthologous groups of CDS were extracted with custom Python scripts via “Gene ID” from the CDS datasets predicted by TransDecoder v5.5.0. Multiple sequence alignments were performed for each orthologous group using MUSCLE v. 3.8.31 [54] with default parameters. The program Gblocks v. 0.9.1 [55] was used to trim alignments with default parameters except for use of the “with-half” gap positions option. Multiple-alignments were concatenated by the program Phyutility v. 2.2.6 [56] to yield the supermatrix used for downstream analyses. The steps of these independent scripts analyses were carried out using previously published Python packages [57].

### 4.4. Phylogenomic Reconstruction of Arundo Species with Nuclear Genes

The 144 matrixes of nuclear OGs with one gene copy per species were subjected to model selection with ModelOMatic [58] to determine whether codon, DNA or amino acid models would fit better the phylogenomic matrix. ModelOMatic analyses were performed with automatic reconstruction of the bionj tree (tree option: “bionj”), standard genetic code (genetic code option: “0”) and full optimization (optimization option: “normal”). In case of program failure, we repeated the run with user-defined trees constructed by ML with PhyML 3.0 using the best model selected by the SMS program [58], either alone or in combination with the “fast” optimization. As in all successful analyses of ModelOMatic codon models resulted in the best-fitting ones, both phylogenies of the single genes and the phylogenomic reconstruction for the whole dataset were conducted with IQ-TREE v. 1.6.8 [58] using codon models. The program ModelFinder [59] implemented in IQ-TREE was used to identify the best model for each of the 144 genes in the dataset using the Bayesian information criterion (BIC) score and to reconstruct the corresponding phylogenetic trees with 1000 replicates of ultrafast bootstrap (UFBOOT [60]). The setting used was “-st CODON1 -m MFP -bb 1000”. For phylogenomic reconstruction, the program ModelFinder implemented in IQ-TREE was used to identify the best partitioning scheme of the whole supermatrix using the fast relaxed clustering algorithm [61,62] by sampling the top 10% partition schemes using edge-linked branch lengths between partitions (option –spp). The best-fit partition model identified was directly used for phylogenomic inference of the species tree by maximum likelihood (ML) with 1000 replicates of ultrafast bootstrapping carried out by resampling sites within partitions. All bootstrap trees generated were written to file with branch lengths. The setting used was “-st CODON1 -m MFP-MERGE -rclusterf 10 -bb 1000 -wbtl”. Gene concordance factor (gCF) and site concordance factor (sCF) were calculated in IQ-TREE v. 1.7 -beta6 by using the previously inferred ML single-gene trees and species supermatrix tree by randomly sampling 100 quartets around each internal branch during sCF computation (option “—scf 100”). Phylogenetic networks were calculated with the SplitsTree4 (v. 4.15.1 [63]) program using the optimal GTR+G+I model on either the whole dataset with base frequencies (A = 0.28; C = 0.21; G = 0.26; T = 0.25), proportion of invariant sites (0.6547) and gamma (0.5558) or the partition constituted by first and second codon positions with base frequencies (A = 0.30; C = 0.21; G = 0.26; T = 0.23), proportion of invariant sites (0.7974) and gamma (0.5754) as calculated by optimal model selection in MEGA X [64]. In addition to the supermatrix approach with IQ-TREE, we also carried out a coalescent supertree approach using ASTRAL III [65] to seek the species tree maximizing the number of quartet trees shared by the single-gene trees reconstructed by IQ-TREE. The option used was “-x” to carry out the exact version of ASTRAL and “-t 2” to output fully annotated branches of the inferred species tree. Maximum parsimony trees were estimated in MEGA X using the Subtree–Pruning–Regrafting (SPR) algorithm with search level 1 in which the initial trees were obtained by random addition of sequences (10 replicates) and 1000 nonparametric bootstrap replicates.

### 4.5. Phylogenetic Reconstruction of Arundinoideae Using the Plastidial Dataset

The chloroplast dataset was constituted by the 5 intergenic regions used in previous studies [2,4,11,66], to which we added the corresponding sequences for the ingroup taxa used for transcriptomics (*A. formosana*, *A. donax* accessions, *A. donaciformis*, *A. micrantha*, *A. plinii* accessions; Table S8). Single genes were aligned with the MAFFT v. 7 online service using automatic method selection [67]. The concatenated alignment, encompassing a total of 4308 aligned nucleotide positions, was used for phylogenetic reconstruction with IQ-TREE v. 1.6.8 [68] analogously to what described above for the nuclear dataset but identifying best DNA models for each gene partition and best-fitting partitioning scheme using the exact search method implemented in ModelFinder [59] and hill-climbing nearest neighbor interchange (NNI) optimization of each bootstrap tree. The options used were “-m MFP-MERGE -bb 1000 -bnni”.

### 4.6. Molecular Dating

Divergence times were estimated using both the nuclear and the plastidial datasets described above using BEAST2 [69]. According to model selection (see below), for both datasets, an uncorrelated lognormal relaxed clock (not considering autocorrelations between adjacent branches [70]) coupled with a Yule demographic prior was used. Substitutions were modeled using the GTR replacement model with four discrete categories of the gamma distribution. For all datasets and analyses, the MCMC was run for 200,000,000 generations, and checked for convergence using Tracer 1.7 [71] to ensure that the effective sample size (ESS) values were greater than 200 for every posterior and for the likelihoods. Consensus trees were obtained by TreeAnnotator 2.5.1 as maximum clade credibility (MCC) trees of all BEAST trees with a burnin of 20% and median heights of nodes.

The nuclear tree was calibrated with the substitution rate previously estimated for Poaceae [58] using a normal distribution to cover between 6 × 10^−3^ and 7 × 10^−3^ (mean 6.5 × 10^−3^; SD 5 × 10^−4^) substitutons/site/million year. The root (*Arundo* stem) was further calibrated with a maximum of 40 MY (million years) that corresponds to the minimum for the origin of PACMAD clade [58]. Because we used a mutation rate previously inferred from four-fold degenerate sites [58], in our analysis, we split our nucleotide alignment into two different partitions: one containing the four-fold degenerate sites which were calibrated using the rate prior; the other containing all other nucleotides [58] which were left free to be estimated. 

To determine which clock and demographic prior fit better the nuclear dataset, the BEAST2 model comparison package [72] was used employing different statistics: AICm (Akaike Information Criterion model), hMean (harmonic mean) and SS (Stepping Stone). The latter was needed because AICm, hMean and BF can be affected by systematic error [73,74].

Three calibration priors were used for the plastid dataset. Previous molecular dating studies have estimated the origin of Poales to be between 120–175 MYA [58]: there is no fossil evidence that place the monocots in this age range [75], but we used 125 MY as a lower bound (maximum) for the BOP-PACMAD stem as in Burke [76,77]. The phytolites (fossil evidence) attributed to the Oryzoideae subfamily [78] were used to calibrate the Oryzoidae stem at 66 MY (upper bound or minimum). As a general prior for all branches the plastidial rate inferred by Christin and colleagues was used [58]: 1× 10^−3^ with a lognormal distribution that ranges between 4.5 × 10^−3^ and 1.2 × 10^−4^ with a median of 6.0 × 10^−4^ substitutions/site/million year. 

### 4.7. Inference of Chromosome-Number Change

To infer chromosome numbers evolution and ploidy levels (diploid or polyploid) for *Arundo* taxa the program ChromEvol v. 2.0 [58] was used. Chromosome numbers frequencies were calculated from the Chromosome Counts Database (CCDB, version 1.45) of plant chromosome numbers (http://ccdb.tau.ac.il/home/) [31] for *M. caerulea*, *H. macra*, *P. australis* and *A. formosana*, while chromosome numbers frequencies for the remaining *Arundo* species were obtained from the literature ([10]; Table S14). The MCC tree obtained from the best model in BEAST2 was used as reference topology (guide tree) for the species tree. The tree was pruned with Newick Utilities v. 1.6 [79] to remove duplicated accessions from the same species (ac: *A. plinii* RO and ama: *A. donax* var. *macrophylla*). The best-fitting among the 10 models implemented in ChromEvol was selected by the Akaike information criterion (AIC) using 6 as base chromosome number (option “_baseNum 6” [10,80]. The optimal chromosome-number evolutionary model was then used to infer the reliability of estimated ploidy levels by using a simulation-based approach on 100 BEAST2 trees randomly sampled from the 16,000 trees retained after burning and pruned with Newick Utilities. 

### 4.8. Molecular Evolution Analyses

Molecular evolution analyses were performed with the HyPhy package v2.2.4 [81]. MACSE [82] was used for generating multiple sequence alignments of each CDS orthologous group. After removing stop codons and sites containing indels, orthologs were assessed for recombination with the GARD method [83], which is implemented in the HyPhy package and can find all the recombination breakpoints, fitting various models using the Akaike Information Criterion (AIC) and Shimodaira-Hasegawa test (SH test) for phylogenetic incongruence. The rate variation was implemented by a general discrete distribution algorithm. Evidence of episodic positive selection was verified by BUSTED [84] (implemented in HyPhy), which uses the branch-site unrestricted statistical test for episodic diversification by estimating the proportion of selected codons across all branches of the phylogenetic tree. The universal genetic code was used. A false-discovery rate (5% FDR) correction was applied to reduce false positives. Detection of episodic diversifying selection for specific branches in the phylogenetic trees was carried out by the adaptive branch-site random effects likelihood (aBSREL) [85] model (implemented in HyPhy), using the universal genetic code. We used the RELAX program [86] implemented in HyPhy for detecting relaxed or intensified selection in the (test) branches identified as under episodic diversifying selection by aBSREL compared to all remaining (reference) branches. Tests of episodic diversifying selection at the level of individual sites were carried out with universal genetic code by the Mixed Effects Model of Evolution (MEME; *p*-value, <0.05) [87] and Fast Unconstrained Bayesian Approximation (FUBAR; posterior probability, ≥0.9) [88] programs, which are implemented in HyPhy. 

Functional annotation of the genes under positive selection was carried out by performing local BLAST searches against the *Arabidopsis thaliana* Araport11 Official Release (06/2016) protein database [89].

## Figures and Tables

**Table 1:** Likelihood and AIC scores for the data set analyzed for each model carried out by the ChromEvol software. ΔAIC is the AIC score difference between each model and the best-fitting one.

MODEL	Parameters	Ln(Likelihood)	AIC	ΔAIC
CONST_RATE_DEMI_EST *	4	−14.92	37.83	0.00
CONST_RATE_DEMI	3	−17.83	41.67	3.83
BASE_NUM	4	−20.30	48.61	10.77
BASE_NUM_DUPL	5	−20.18	50.36	12.53
CONST_RATE	3	−29.81	65.61	27.78
CONST_RATE_NO_DUPL	2	−31.15	66.30	28.47
LINEAR_RATE_DEMI	5	−29.61	69.22	31.39
LINEAR_RATE	5	−29.99	69.98	32.14
LINEAR_RATE_NO_DUPL	4	−31.02	70.03	32.20
LINEAR_RATE_DEMI_EST	6	−29.49	70.98	33.14

*: best evolutionary model.

**Table 2:** Homology-based functional annotation of the 7 genes under positive selection after multiple test correction (FDR: 0.05) according to the sequence alignment-wide method implemented in BUSTED.

OG ID	Sites	LR	*p*-Value	Fdr	TAIR_ID	Name	Description
OG0018377	1531	39.72	2.38 × 10^−9^	2.88 × 10^−7^	AT5G47690	*PDS5A*	PO76/PDS5 cohesin cofactor
OG0018357	964	29.35	4.23 × 10^−7^	3.05 × 10^−5^	AT4G34100	*CER9*	RING/U-box-superfamily-protein
OG0018102	258	22.17	1.53 × 10^−5^	0.000735	AT5G48300	*ADG1*	ADP glucose pyrophosphorylase 1
OG0018342	973	17.99	0.000124	0.004471	AT4G11420	*EIF3A*	Eukaryotic translation initiation factor 3 subunit A
OG0018364	899	13.89	0.000965	0.027788	AT4G16150	*CAMTA5*	Calmodulin-binding; transcription-regulators
OG0018157	445	12.44	0.001986	0.047669	AT4G35140	NA	Transducin/WD40-repeat-like-superfamily-protein
OG0018070	126	12.10	0.002359	0.048518	NA	NA	NA

**Table 3:** Homology-based functional annotation of the 7 genes undergoing branch-specific selection intensification after multiple test corrections (FDR: 0.05) according to ABSREL and RELAX. The higher the K value, the higher the predicted entity of selection intensification. Node name abbreviations: adf, *A. donaciformis*; ama, *A. donax* var. *macrophylla*; ami, *A. micrantha*; hm, *H. macra*; mc, *M. caerulea*; pa, *P. australis*.

OG ID	Test Nodes	K	LR	FDR *p*-Val ^a^	TAIR ID	Name	Description
OG0017620	hm	4.35	6.19	0.030	AT5G19210	NA	P-loop containing nucleoside triphosphate hydrolases superfamily protein
OG0018017	ami	3.69	10.02	0.014	AT1G64710	NA	GroES-like zinc-binding alcohol dehydrogenase family protein
OG0018069	adf	39.10	8.39	0.014	AT5G42020	*BIP2*	Luminal binding protein involved in polar nuclei fusion during proliferation of endosperm nuclei.
OG0018170	pa	3.79	9.98	0.009	AT2G01680	NA	Ankyrin repeat family protein
OG0018204	adf	4.73	6.09	0.028	AT5G08530	*CI51*	51 kDa subunit of mitochondrial complex I
OG0018205	mc	9.25	15.13	0	AT3G15140	*ERI-1*	Ribonuclease H-like superfamily functioning as siRNA exonuclease. It affects post-transcriptional gene silencing and growth rate /biomass.
OG0018357 *	ama, mc	3.67	7.93	0.014	AT4G34100	*CER9*	Involved in cuticular wax biosynthesis. Arabidopsis mutants have leaf waxes nearly pure C24 and C26 acid, weakly glaucous stem surface, and reduced fertility in early flowers.

^a^ Benjamini-Hochberg multiple test correction. * genes identified as positively selected according to BUSTED.

## Supplementary Materials

Supplementary materials can be found at https://www.mdpi.com/1422-0067/21/15/5247/s1.

## Abbreviations

MYA	Million years ago
GO	Gene ontology
ML	Maxim likelihood
MP	Maxim parsimony
BI	Bayesian inference
gCF	Gene concordance factor
sCF	Site concordance factor
gEF_p	Probability of equal frequencies rejection for genes
sEF_p	Probability of equal frequencies rejection for genes
gIC	Gene Internode Certainty
sIC	Site Internode Certainty
PP	Posterior probability
UFBOOT	Ultrafast bootstrap
npBS	Nonparametric bootstrap
HPD	High Posterior Densities
