# Supplementary material for: Phylogenomic proof of Recurrent Demipolyploidization and Evolutionary Stalling of the “Triploid Bridge” in Arundo (Poaceae)
Source: Int J Mol Sci. 2020 Jul 24;21(15):5247. doi: 10.3390/ijms21155247 (PMC7432733; doi:10.3390/ijms21155247)
Supplement: Supplementary file 1 [file ijms-21-05247-s001.zip › Suppl_Tables_09-12.docx]

**Supplementary Table 9:** Summary of clock models and priors tested for molecular dating of the *Arundo* genus and selected outgroups divergence based on the nuclear dataset. Chain lenght 200,000,000. Numbers in bold indicate optimal (minimal) values. The best model is highlighted in red.

| ID | Clock | Rate distribution | Demographic prior | Xml file | Likelihood | Posterior | AICm | H_mean | Stepping Stone |
| --- | --- | --- | --- | --- | --- | --- | --- | --- | --- |
|  |  |  |  |  |  |  |  |  |  |
| 1 | Relaxed | LogNormal | Yule | GGRlogY_nr_RlogN | **-403604** | **-403651** | 807262 | -403623 | -403820 |
| 2 | Relaxed | Normal | Yule | GGRlogY_nr_R | **-403604** | **-403651** | **807261** | **-403621** | **-403778** |
| 3 | Relaxed | Normal | Birth and death | GGRlogBD_nr_R | **-403604** | -403658 | **807261** | -403623 | -404302 |
| 4 | Relaxed | Normal | Coal constant | GGRlogCoCo_nr_R | -403636 | -403714 | 807342 | -403658 | -403964 |
| 5 | Strict | Normal | Yule | GGSY_nr_R | -403825 | -403871 | 807693 | -403839 | -403958 |

**Supplementary Table 10**: Summary of clock rates estimated from the nuclear dataset with different models and data partitions. The best model is highlighted in red.

|  | Xml file | Partition | Mean Rate^a^ 10^-3^ | Rate^a^ 95% 10^-3^ | Overall clock^a, b^ | Overall variance 10 ^-7^ | Root age (95% CI) | *Arundo* split (95% CI) |
| --- | --- | --- | --- | --- | --- | --- | --- | --- |
|  |  |  |  |  |  |  |  |  |
| 1 | GGRlogY_nr_RlogN | Not 4 Fold | 1.758 | 1.288 -2.227 | 2.453 | 2.7487 | 17.5 (13.3 -22.5) | 7.8 (5.8 – 9.8) |
|  |  | 4 Fold | 6.621 | 5.401 -7.989 |  |  |  |  |
| 2 GGRlogY_nr_R | | Not 4 Fold | 1.74 | 1.318 - 2.161 | 2.329 | 0.3926 | 17.8 (13.9 – 22.2) | 7.9 (6.2 – 9.8) |
|  |  | 4 Fold | 6.575 | 5.581 - 7.453 |  |  |  |  |
| 3 | GGRlogBD_nr_R | Not 4 Fold | 2.01 | 9.933 - 3.052 | 2.807 | 0.582 | 16.4 (8.9 – 26.4) | 7.3 ( 3.9 – 11.7) |
|  |  | 4 Fold | 7.601 | 3.913 - 11.2 |  |  |  |  |
| 4 | GGRlogCoCo | Not 4 Fold | 220.9 | 158.6 - 287.5 | 19.053 | 799.827 | 39.4 (38.3 – 40) | 8.5 (7.8 – 9.3) |
|  |  | 4 Fold | 8.539 | 7.866 - 9.227 |  |  |  |  |
| 5 | GGSY_nr_R | Not 4 Fold | 1.561 | 1.235 - 1.882 | 2.278 | 0.2498 | 18 (14.3 – 21.8) | 7.8 (6.1 – 9.4) |
|  |  | 4 Fold | 6.58 | 5.573 - 7.523 |  |  |  |  |

^a^ the clock rate is calculated in substitutons/site/million year;

^b^ the overall clock is calculated using the weighted mean of the two partitions as follows: *weighted clock* $\boldsymbol{C=}\sum_{\boldsymbol{i=1}}^{\boldsymbol{n}} \boldsymbol{\omega}_{\boldsymbol{i}}^{\boldsymbol{'}}\boldsymbol{c}_{\boldsymbol{i}}$ , where $\boldsymbol{c}_{\boldsymbol{i}}$ are the estimated mean rates for the 4-fold degenerate and non-degenerate partitions and $\boldsymbol{\omega}_{\boldsymbol{i}}\boldsymbol{=}\frac{\boldsymbol{No. of partition site}}{\boldsymbol{Total No. of site}}$ are the respective normalized weights.

^c^ the overall variance is calculated using the weighted variance of the two partitions as follows: *weighted variance* $\boldsymbol{\sigma}_{\boldsymbol{C}}^{\boldsymbol{2}}\boldsymbol{=}\sum_{\boldsymbol{i=1}}^{\boldsymbol{n}} \boldsymbol{\omega}_{\boldsymbol{i}}^{\boldsymbol{'2}}\boldsymbol{\sigma}_{\boldsymbol{c}_{\boldsymbol{i}}}^{\boldsymbol{2}}$, where$\boldsymbol{\sigma}_{\boldsymbol{c}_{\boldsymbol{i}}}^{\boldsymbol{2}}$ are the estimated mean rates for the 4-fold degenerate and non-degenerate partitions and $\boldsymbol{\omega}_{\boldsymbol{i}}\boldsymbol{=}\frac{\boldsymbol{No. of partition codons}}{\boldsymbol{Total No. of codons}}$ are the respective normalized weights.

**Supplementary Table 11**: Summary of ages for *Arundo* clades. Mean age and 95% confidence intervals were estimated from the nuclear dataset with the best fitting model. Node color-coding refers to Fig. 4 in the main text.

| Node | Node name | Mean age (Million of years) | 95 % C.I (Million of years) |
| --- | --- | --- | --- |
|  |  |  |  |
|  | *Arundo* origin | 17.8 | 13.9 -22.2 |
|  | *Arundo* split (*A. formosana* split) | 7.9 | 6.2 – 9.8 |
|  | *A. donax* split | 5.7 | 4.5 _ 7.1 |
|  | *A. micrantha* split | 4.7 | 3.8 – 5.9 |
|  | *A. donax* varieties split | 3.6 | 2.6 -4.7 |
|  | *A. plinii s. l.* crown | 3.4 | 2.6 -4.3 |
|  | *A. donaciformis* split | 2.4 | 1.8 -3.1 |
|  |  |  |  |
|  |  |  |  |

**Supplementary Table 12**: Summary of ages for BOP-PACMAD and Arundinoideae clades. Mean age and 95% confidence intervals were estimated from the chloroplast dataset with the best fitting model. Node IDs (letters) refer to Suppl. Fig. XX.

| Node | Node name | Mean age (Million of years) | 95 % C.I (Million of years) |
| --- | --- | --- | --- |
|  |  |  |  |
| 0 | BOP –PACMAD origin | 97.9 | 76 -124 |
| A | BOP-PACMAD split | 90.4 | 73 - 113 |
| B | BOP | 82.5 | 69 -100 |
| C | PACMAD | 68.7 | 47 -90 |
| D | Arundinoidea origin | 51.8 | 34 -70 |
| E | Arundinoidea split | 47.4 | 30 -64 |
| F | *Arundo* origin | 29.3 | 14-43 |
| G | *Arundo* split | 9.7 | 7.5 - 11 |
