# Supplementary figures and images for: Phylogenomic proof of Recurrent Demipolyploidization and Evolutionary Stalling of the “Triploid Bridge” in Arundo (Poaceae)
Source: Int J Mol Sci. 2020 Jul 24;21(15):5247. doi: 10.3390/ijms21155247 (PMC7432733; doi:10.3390/ijms21155247)

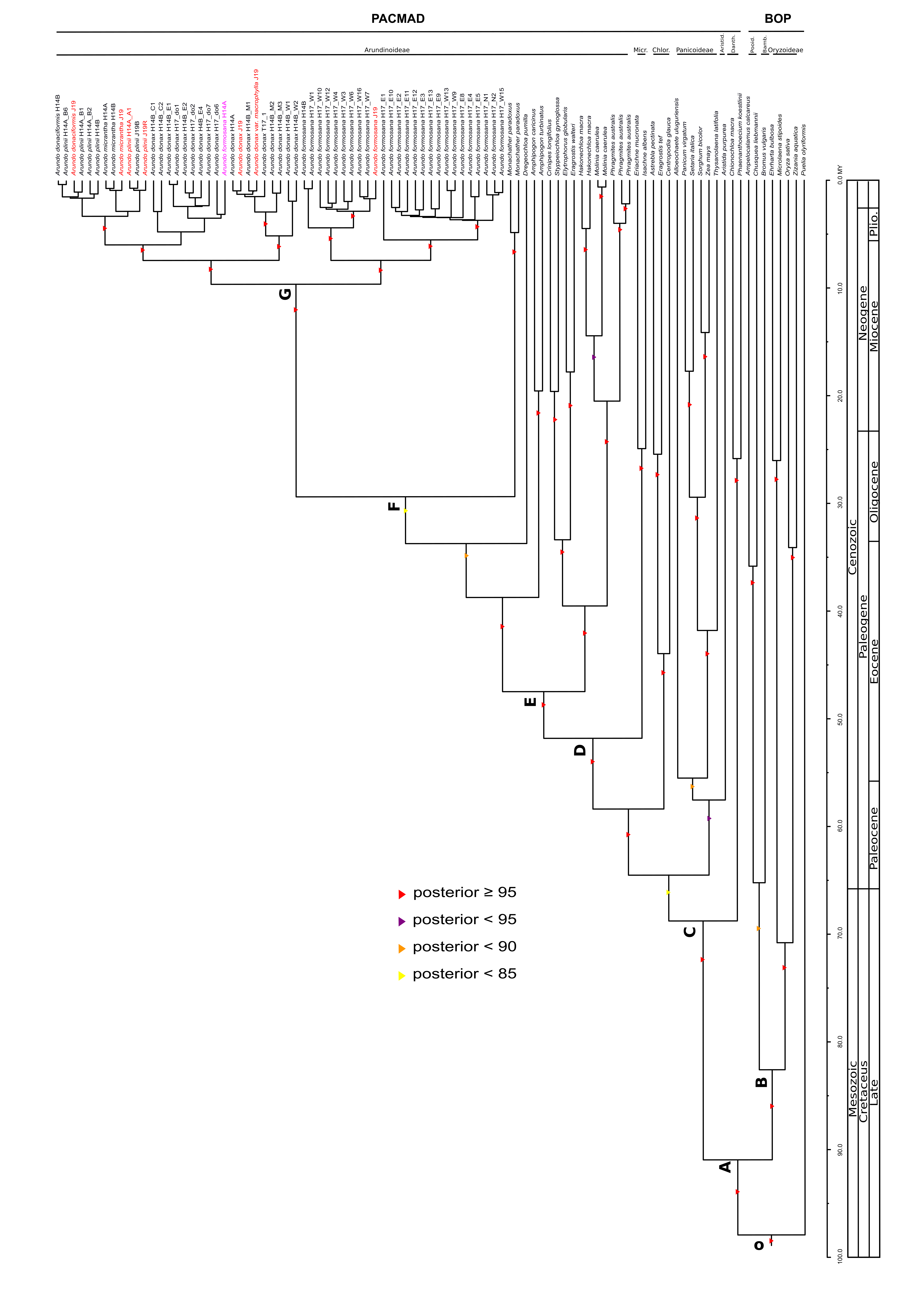

Supplement: Supplementary file 1 [file ijms-21-05247-s001.zip › Suppl_Fig_1.png]
